# *Lycium barbarum* Glycopeptide Alleviates Neomycin‐Induced Ototoxicity by Inhibiting Tryptophan Hydroxylase‐Mediated Serotonin Biosynthesis

**DOI:** 10.1002/advs.202405850

**Published:** 2025-03-26

**Authors:** Yunhao Wu, Li Zhang, Shengda Cao, Jingwen Zhang, Cheng Li, Yunlong Shan, Qiuping Liu, Zhexiong Yu, Qiaojun Fang, Yuhua Zhang, Xiaolong Fu, Kwok‐Fai So, Renjie Chai

**Affiliations:** ^1^ Medical Science and Technology Innovation Center Shandong First Medical University & Shandong Academy of Medical Sciences Jinan 250000 China; ^2^ State Key Laboratory of Digital Medical Engineering Department of Otolaryngology Head and Neck Surgery Zhongda Hospital School of Life Sciences and Technology Advanced Institute for Life and Health Jiangsu Province High‐Tech Key Laboratory for Bio‐Medical Research Southeast University Nanjing 210096 China; ^3^ Key Laboratory of CNS Regeneration (Ministry of Education) Guangdong‐Hong Kong‐Macau Institute of CNS Regeneration Jinan University Guangzhou 510632 China; ^4^ Department of Otorhinolaryngology Qilu Hospital of Shandong University NHC Key Laboratory of Otorhinolaryngology (Shandong University) Jinan 250063 China; ^5^ Department of Otolaryngology‐Head and Neck The Second Affiliated Hospital of Guangzhou University of Chinese Medicine Guangzhou 510120 China; ^6^ Yixing Traditional Chinese Medicine Hospital Yixing 214299 China; ^7^ Key Laboratory of Drug Metabolism and Pharmacokinetics State Key Laboratory of Natural Medicines China Pharmaceutical University Nanjing 210009 China; ^8^ Department of Otolaryngology‐Head and Neck Surgery Affiliated Hospital of Jiangnan University Wuxi 214000 China; ^9^ Tianren Goji Biotechnology Co., Ltd Ningxia 755000 China; ^10^ Department of Otolaryngology‐Head and Neck Surgery the Second Affiliated Hospital of Anhui Medical University Hefei 230601 China; ^11^ Guangdong‐Hongkong‐Macau Institute of Central Nervous System (CNS) Regeneration Ministry of Education Central Nervous System (CNS) Regeneration Collaborative Joint Laboratory Jinan University Guangzhou 510632 China; ^12^ Co‐Innovation Center of Neuroregeneration Nantong University Nantong 226001 China; ^13^ School of Medical Technology Institute of Engineering Medicine Beijing Institute of Technology Beijing 100081 China; ^14^ Department of Otolaryngology Head and Neck Surgery Sichuan Provincial People's Hospital University of Electronic Science and Technology of China Chengdu 611731 China; ^15^ Institute for Stem Cell and Regeneration Chinese Academy of Science Beijing 100101 China

**Keywords:** *Lycium barbarum* glycopeptide, P‐chlorophenylalanine, sensorineural hearing loss, tryptophan hydroxylase, tryptophan metabolism

## Abstract

Aminoglycoside antibiotic‐induced sensorineural hearing loss (SNHL) is a common sensory disorder that requires the development of prophylactic and therapeutic interventions. *Lycium barbarum* glycopeptide (LBGP) is a peptidoglycan isolated and purified from *Lycium barbarum* polysaccharides that exhibit significant anti‐inflammatory, antioxidant, and neuroprotective effects, but the role of LBGP in aminoglycoside‐induced SNHL has not been well investigated. Here it is shown that LBGP can protect against neomycin‐induced hearing impairment and alleviate oxidative stress in a neomycin‐induced SNHL mouse model. Moreover, it is further found that inhibition of tryptophan hydroxylase (Tph)‐mediated serotonin (5‐HT) biosynthesis plays a key role in the mechanism of action of LBGP in treating neomycin‐induced hearing loss. Systemic delivery of 5‐HT increased neomycin‐induced apoptosis of cochlear hair cells and spiral ganglion neurons, and pharmacological Tph2 inhibition with P‐chlorophenylalanine or *Tph2* knock down by AAV‐ie‐*Tph2* effectively attenuated neomycin‐induced hearing dysfunction. Collectively, these results provide a promising strategy for the prevention of SNHL by using natural plant extract which is more available and exhibits lower side effects compared with other otoprotective drugs, and identify Tph2 as a potential pharmacological target for the treatment of aminoglycoside‐induced ototoxicity.

## Graphical abstract

*Lycium barbarum* glycopeptide (LBGP), which is further extracted from *Lycium barbarum* polysaccharides, exhibits significant protective effects against neomycin‐induced hearing dysfunction including oxidative stress in cochlea and loss of key cells in cochlea. The underlying mechanism of the otoprotective effect of LBGP may be associated with the inhibition of tryptophan hydroxylase‐mediated 5‐HT biosynthesis.

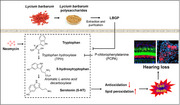

## 1 Introduction

Aminoglycoside antibiotics are formed through the connection of amino sugars and aminocyclic alcohols by oxygen bridges, and they are used extensively for treating bacterial infections in the clinic. However, a growing body of evidence has demonstrated that aminoglycosides application can lead to irreversible hearing loss by inducing oxidative stress and apoptosis in cochlear cells.^[^ ^1^, ^2^, ^3^ ^]^ Although alternative antibiotics have been developed, aminoglycosides are still used due to their high efficacy, low cost of production, and widespread availability. It is thus vital to explore therapeutic medications to address aminoglycoside‐induced hearing loss.

*Lycium barbarum*, also known as wolfberry, is widely distributed in China, and its fruits have been used as a traditional Chinese medicine with nourishing effects for thousands of years.^[^ ^4^ ^]^ *Lycium barbarum* polysaccharides are the main bioactive components of *Lycium barbarum*, and they exert significant antioxidant, anti‐inflammatory, immunomodulatory, and neuroprotective effects.^[^ ^5^, ^6^, ^7^, ^8^ ^]^ *Lycium barbarum* glycopeptide (LBGP), which is further extracted from *Lycium barbarum* polysaccharides, is a glycoconjugate that consists of monosaccharides (such as arabinose, glucose, and galactose) and 30% protein.^[^ ^9^ ^]^ LBGP is considered to be the most valuable and potent component of *Lycium barbarum* fruits and has high medicinal values for improving anxiety disorders, virus infection, colitis, and glioma.^[^ ^9^, ^10^, ^11^, ^12^ ^]^ *Lycium barbarum* exhibits improvement on hearing function according to the recordation in ancient medical books and Chinese pharmacopoeia in China. It has been reported that *Lycium barbarum* polysaccharides can effectively attenuate cisplatin‐induced accumulation of reactive oxygen species (ROS) and lower the mitochondrial membrane potential in organ of Corti explants, thus further protecting against hair cell loss.^[^ ^13^ ^]^ However, whether LBGP can prevent aminoglycoside‐induced hearing loss remains poorly understood.

In the current study, we evaluated the otoprotective effect of LBGP against neomycin‐triggered hearing dysfunction through audiometry and immunostaining of cochlear hair cells, spiral ganglion neurons (SGNs), neurofilaments and ribbon synapses, and investigated the mechanism underlying the action of LBGP on hearing loss by untargeted metabolomics analysis.

## 2 Results

### 2.1 LBGP Prevents Loss of Cochlear Hair Cells and SGNs after Neomycin Exposure

**Figure 1:**
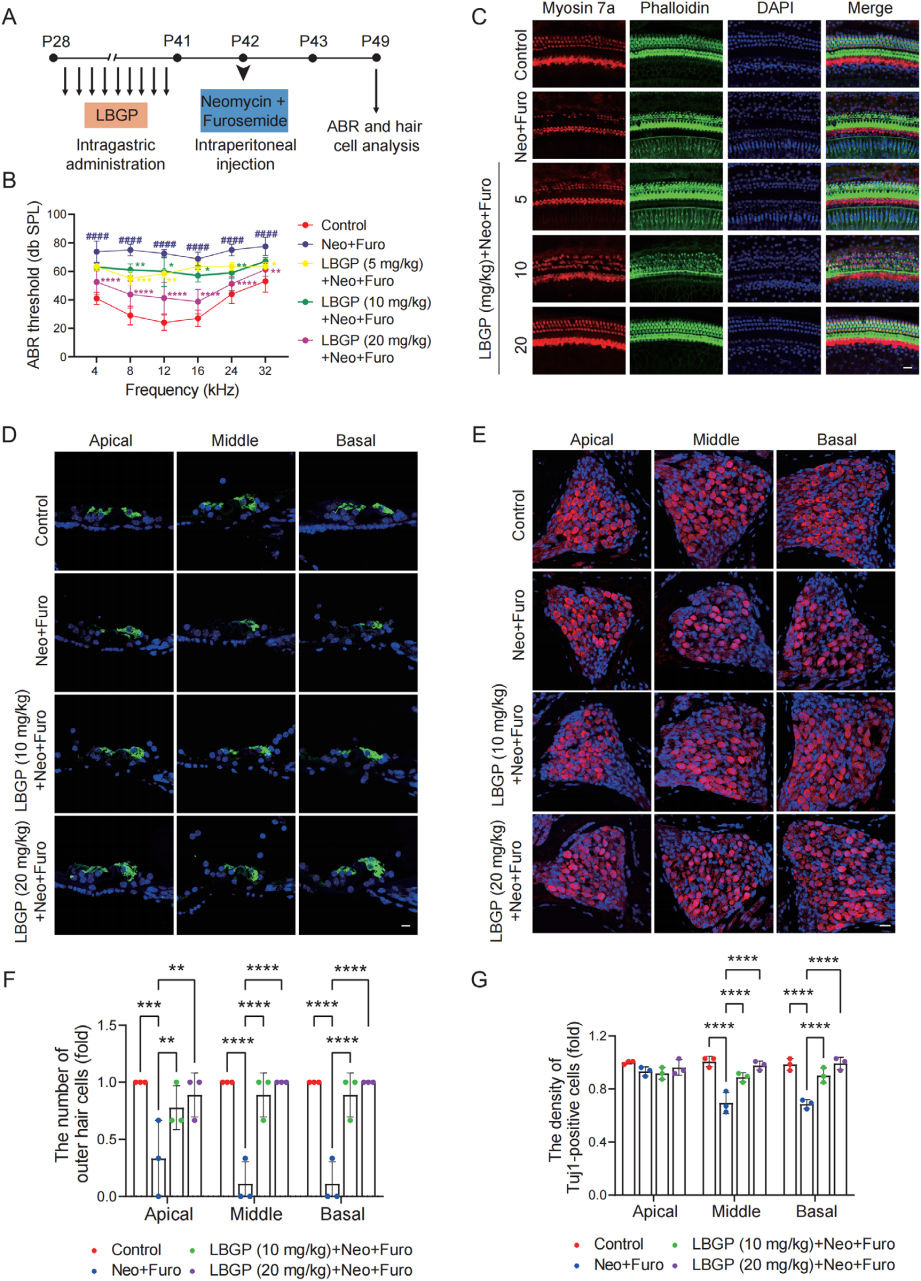
LBGP prevents neomycin‐induced hearing dysfunction in C57BL/6 mice. A) Schematic illustration of the experimental design. B) ABR thresholds of mice in different groups (*n* = 6). Results are presented as mean ± SD, ^####^ *P* < 0.0001 versus Control group; *P < 0.05, **P < 0.01, ***P < 0.001, ****P < 0.0001 versus Neo+Furo group by two‐way ANOVA with Bonferroni post hoc test. C) Immunostaining of cochlear hair cells with anti‐myosin 7a, phalloidin, and DAPI (*n* = 3). Scale bar, 20 µm. D) Immunofluorescence analysis of myosin 7a in frozen section of mouse cochleae (*n* = 3). Scale bar, 10 µm. E) Immunofluorescence analysis of Tuj1 in frozen sections of mouse cochleae (*n* = 3). Scale bar, 20 µm. F,G) The relative number of outer hair cells or SGNs in the different groups (*n* = 3). Data are presented as mean ± SD, **P < 0.01, ***P < 0.001, ****P < 0.0001 by two‐way ANOVA with Bonferroni post hoc test.

The structure and the high‐performance liquid chromatography (HPLC) profiles of LBGP was shown in Figure S1 (Supporting Information). In order to investigate the impact of LBGP on hearing impairment, we established an acute model of neomycin‐induced ototoxicity (**Figure** **1**A). C57BL/6 mice were given LBGP (5, 10, or 20 mg kg^−1^) daily by intragastric administration for 2 weeks. The acute hearing loss model of C57BL/6 mice was then established by intraperitoneal injection of neomycin at 100 mg kg^−1^ followed by the intraperitoneal injection of 200 mg kg^−1^ furosemide 1 h later. The results of auditory brainstem response (ABR) indicated that LBGP at 5, 10, 20 mg kg^−1^ reduced neomycin‐induced elevated thresholds, and LBGP at 20 mg kg^−1^ exhibited the best protective effect (Figure 1B). The number of surviving myosin 7a‐positive hair cells was significantly decreased following neomycin treatment in comparison to the control group. However, the pre‐treatment of LBGP demonstrated a significant reduction in neomycin‐induced cochlear hair cell loss (Figure 1C, Figure S2, Supporting Information). And the result of cochlear frozen sections was consistent with the above findings (Figure 1D,F). SGNs were then stained with Tuj1 and the quantitative analysis showed that Tuj1‐positive SGNs were significantly reduced in the middle and basal turns in the neomycin group. However, the LBGP treatment group exhibited remarkably higher numbers of surviving SGNs (Figure 1E,G). These results suggested that oral administration of LBGP could effectively ameliorate neomycin‐induced hearing loss in vivo.

### 2.2 LBGP Attenuates Neomycin‐Induced Degeneration of Cochlear Neurofilaments and Ribbon Synapses

**Figure 2:**
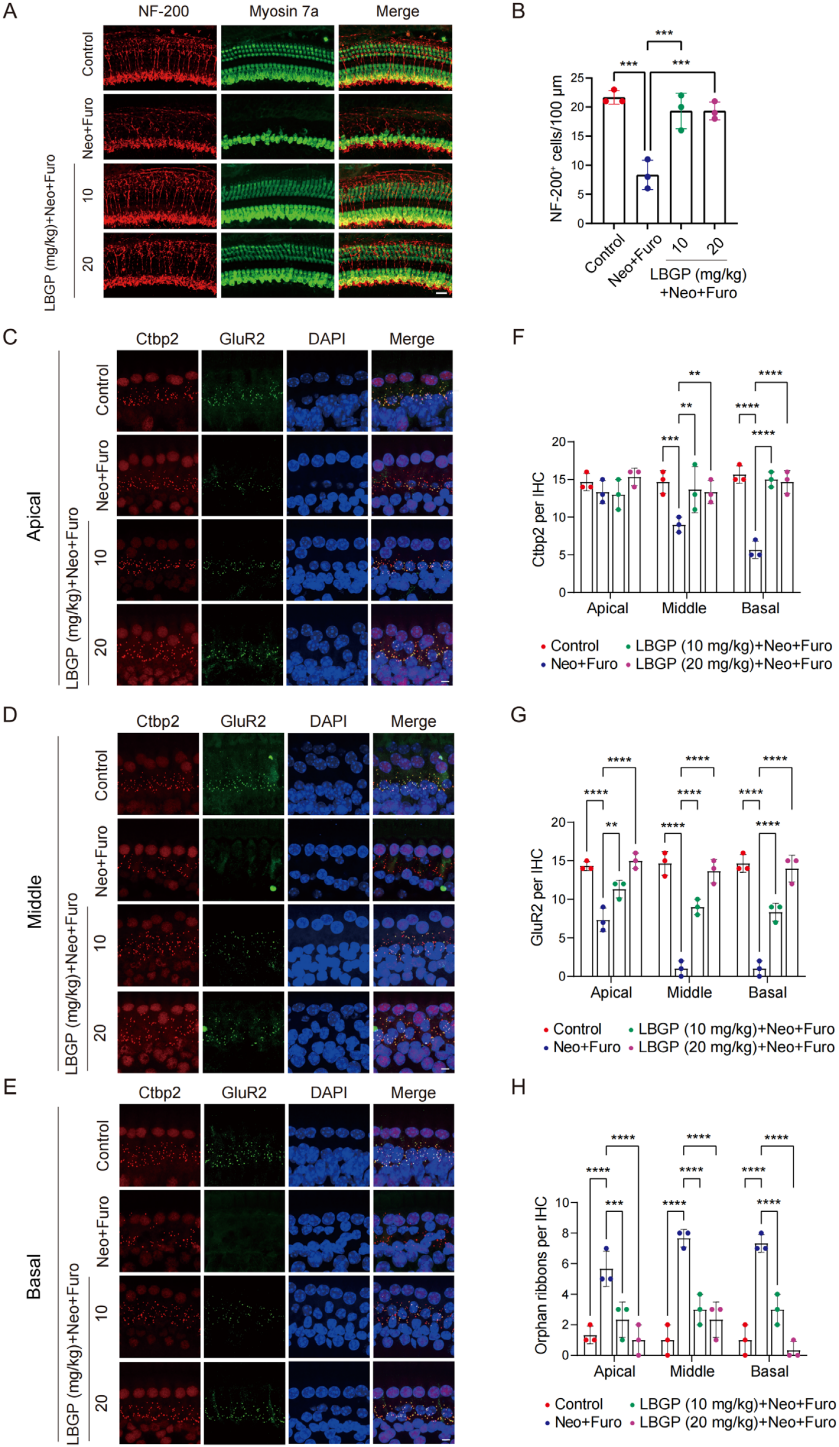
LBGP rescues neomycin‐induced loss of ribbon synapses in C57BL/6 mice. A) Immunostaining with NF‐200 (a maker of neurofilaments) in the middle turns of cochleae from the different groups (*n* = 3). Scale bar, 20 µm. B) Quantification of NF‐200‐positive cells in the different groups. Results are presented as mean ± SD, ***P < 0.001 by one‐way ANOVA with Bonferroni post hoc test. C–E) Immunostaining for Ctbp2 and GluR2 in the apical, middle, and basal turns of cochleae in the different groups (*n* = 3). Scale bar, 5 µm. F–H) Quantification of Ctbp2, GluR2, and Orphan ribbons. Results are presented as mean ± SD, **P < 0.01, ***P < 0.001, ****P < 0.0001 by two‐way ANOVA with Bonferroni post hoc test.

We next explored the role of LBGP in maintaining adequate cochlear innervation. As shown in **Figure** **2**A, there were many auditory nerve axons contacting outer hair cells in the control group, but significant loss of axons from inner hair cells to outer hair cells was observed in the cochlear middle turn in the neomycin group. However, NF‐200‐positive projections were notably more abundant in the LBGP group in comparison to the neomycin‐treated group (Figure 2A,B). To determine whether the loss of neurofilaments resulted in a change in ribbon synapses, the cochlear ribbon and postsynaptic receptors were marked with Ctbp2 and Glur2 antibodies (Figure 2C–E) and quantified. After double labeling with these two antibodies, the combined areas showed ribbon synapses.^[^ ^14^ ^]^ The study revealed a notable decrease in the number of ribbon synapses in the middle and basal turns of the cochlea following neomycin‐induced damage. However, pretreatment with LBGP was found to effectively restore the loss of ribbon synapses, as evidenced by the presence of pre‐synaptic dots (Figure 2F) and post‐synaptic puncta (Figure 2G) in the images. When the pre‐synaptic signal disconnects from the post‐synaptic puncta this results in a decrease in the action potential of SGNs, so we also quantified the number of orphan ribbons. We found that LBGP significantly reduced the number of orphan ribbons in the basal turn of the cochlea compared with the neomycin group (Figure 2H). Collectively, these results showed that LBGP protected against neomycin‐induced loss of neuronal fibers and ribbon synapses.

### 2.3 LBGP Inhibits Oxidative Stress Induced by Neomycin Exposure

**Figure 3:**
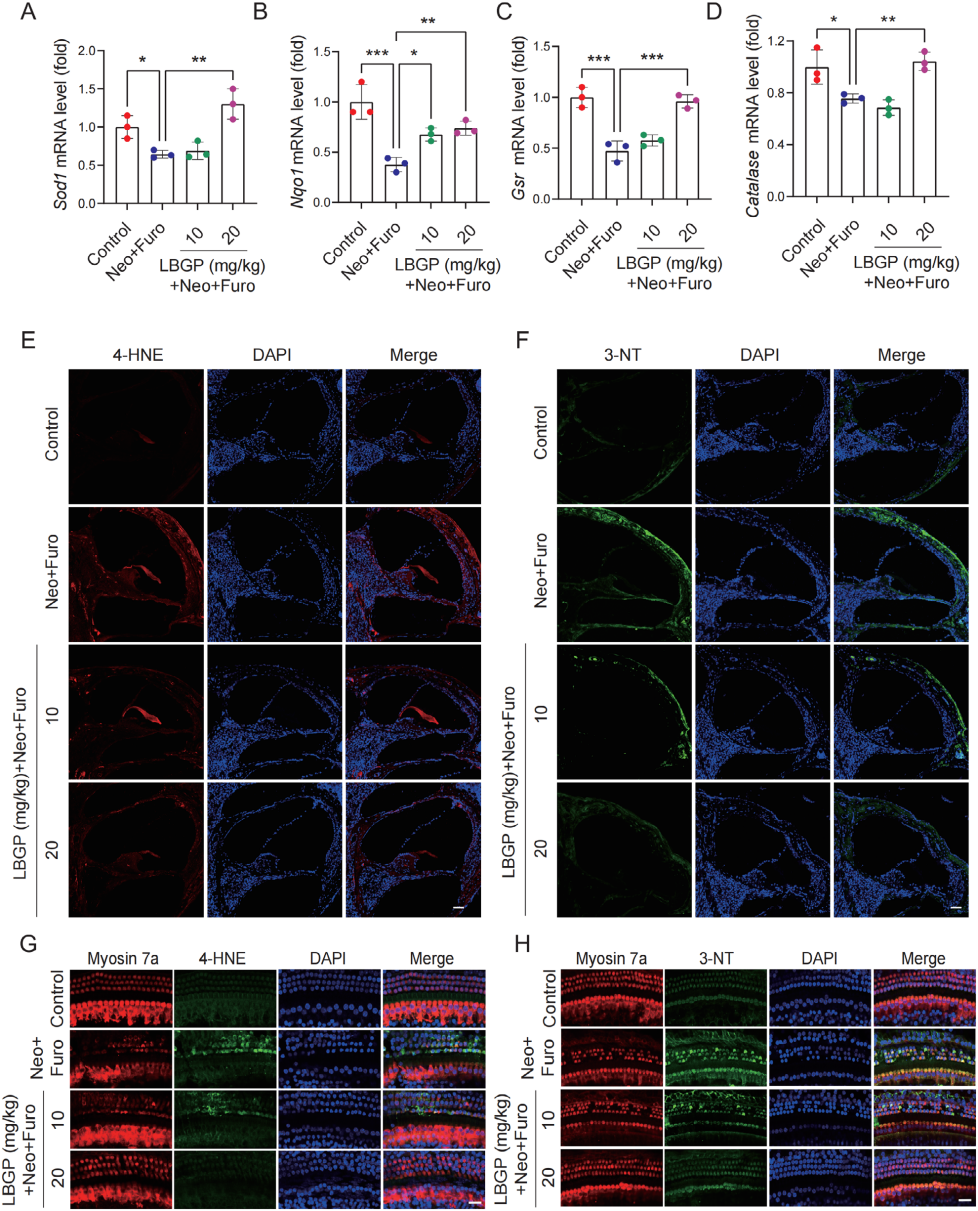
LBGP ameliorates the oxidative stress induced by neomycin in C57BL/6 mice. A–D) The mRNA levels of the antioxidant genes *Sod1*, *Nqo1*, *Gsr*, and *Catalase* were measured by qPCR in the different groups (*n* = 3). E,F) Immunostaining with 4‐HNE and 3‐NT in frozen sections of mouse cochleae in the different groups (*n* = 3). Scale bar, 50 µm. G,H) Immunostaining with 4‐HNE and 3‐NT in cochlear hair cells (stained with Myosin 7a) in the different groups (*n* = 3). Scale bar, 20 µm. Results are presented as mean ± SD, *P < 0.05, **P < 0.01, ***P < 0.001 by one‐way ANOVA with Bonferroni post hoc test.

Studies have indicated that oxidative injury is a critical factor in aminoglycoside‐induced ear damage,^[^ ^15^ ^]^ therefore, our investigation focused on the impact of LBGP on neomycin‐induced redox imbalance. The quantitative real‐time PCR results indicated that the mRNA levels of four vital antioxidant genes (*Sod1*, *Nqo1*, *Gsr*, and *Catalase*) in the mouse cochlea were significantly decreased after neomycin treatment, and that LBGP (20 mg kg^−1^) pretreatment reversed the decreased levels of these genes (**Figure** **3**A–D). Immunostaining results showed that LBGP could effectively decrease neomycin‐triggered elevation of 4‐hydroxynonenal (4‐HNE) and 3‐nitrotyrosine (3‐NT) expression in the cochlea (Figure 3E,F). Consistent with these results, we also observed that the expression levels of 4‐HNE and 3‐NT in hair cells were significantly reduced in the LBGP treatment group compared to the neomycin group (Figure 3G,H). Taken together, these results disclosed that LBGP alleviated neomycin‐induced ototoxicity by inhibiting cochlear oxidative stress.

### 2.4 LBGP Modulates Tryptophan Metabolism and Reduces Neomycin‐Induced 5‐HT Production

**Figure 4:**
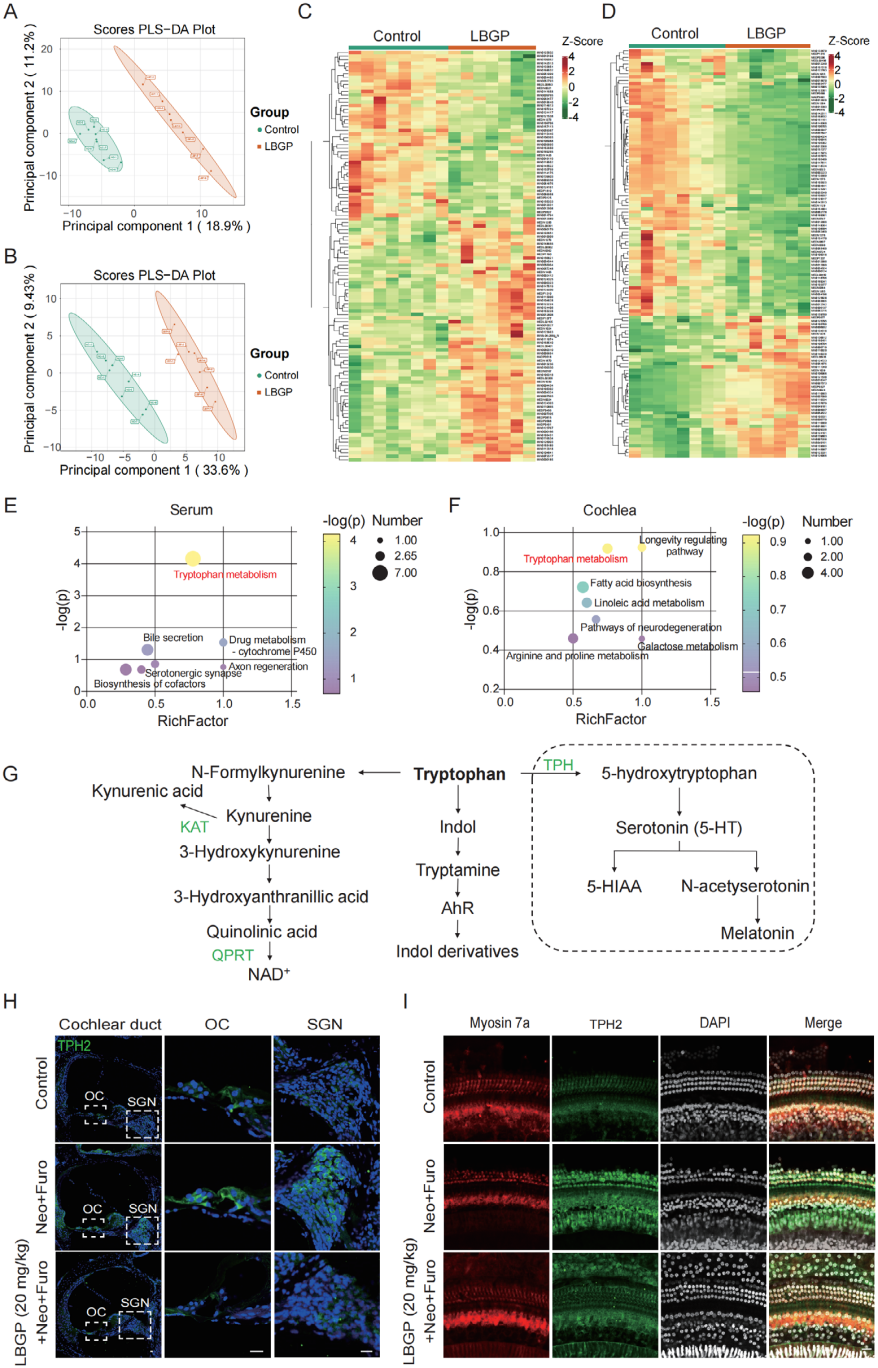
LBGP alters the metabolic profile and inhibits 5‐HT metabolism in C57BL/6 mice. A,B) Partial least squares discriminant analysis (PLS‐DA) of metabolomic profiles in serum and cochleae in the different groups (*n* = 7–8). C,D) Heatmaps of significantly altered metabolites in serum and cochleae in the different groups (*n* = 7–8). E,F) Pathway enrichment analysis of the significantly changed metabolites in serum and cochleae in the different groups. G) Tryptophan is metabolized via three distinct pathways: the kynurenine pathway, the 5‐HT pathway, and the indole pathway. H) Immunostaining with Tph2 in the organ of Corti (OC) and SGNs in the cochlea (*n* = 3). Scale bar, 10 µm. I) Immunostaining with Tph2 in cochlear hair cells (stained with Myosin 7a) (*n* = 3). Scale bar, 20 µm.

Metabolites in the circulatory system have been shown to be involved in neurodegenerative disorders via mediating different signaling pathways.^[^ ^16^ ^]^ In the present study, a non‐targeted metabolomics analysis was performed to identify the critical metabolites or metabolic pathways that altered in the mouse cochlea and serum and thus might be involved in the mechanism of action of LBGP in treating neomycin‐induced hearing loss. The result of partial least squares discriminant analysis exhibited a significant separation of clusters between the control group and the LBGP group in the serum and cochlea (**Figure** **4**A,B). Heatmap analysis showed a remarkable change in metabolites between the control group and LBGP group, with a total of 116 altered metabolites in the serum and 124 altered metabolites in the cochlea (Figure 4C,D). The metabolomic maps exhibited the significantly enriched metabolic pathways that were influenced by LBGP in the serum and the cochleae, among which tryptophan metabolism was strongly influenced by LBGP both in the serum and in the cochlea (Figure 4E,F). Among the pathways that tryptophan was metabolized, 5‐HT pathway played a critical role in the pathogenesis of hearing impairment, and Tph is the rate‐limiting enzyme in the synthesis of 5‐HT in tryptophan metabolism (Figure 4G). Due to there exist two isoforms of Tph (Tph1 and Tph2), we first detected the expression of Tph1 in the mouse cochlea and found that there was no expression of Tph1 in the cochlea (Figure S3, Supporting Information). We further measured the expression of Tph2 in the mouse cochlea and found that neomycin treatment induced elevated expression of Tph2 in cochlear hair cells and SGNs, while there was an inhibition of Tph2 expression in the LBGP group (Figure 4H,I). These results indicated that inhibition of Tph2‐mediated 5‐HT biosynthesis might play a significant role in LBGP‐mediated protection against neomycin‐induced hearing loss.

### 2.5 Systemic Delivery of 5‐HT Aggravates Neomycin‐Induced Hearing Impairment

**Figure 5:**
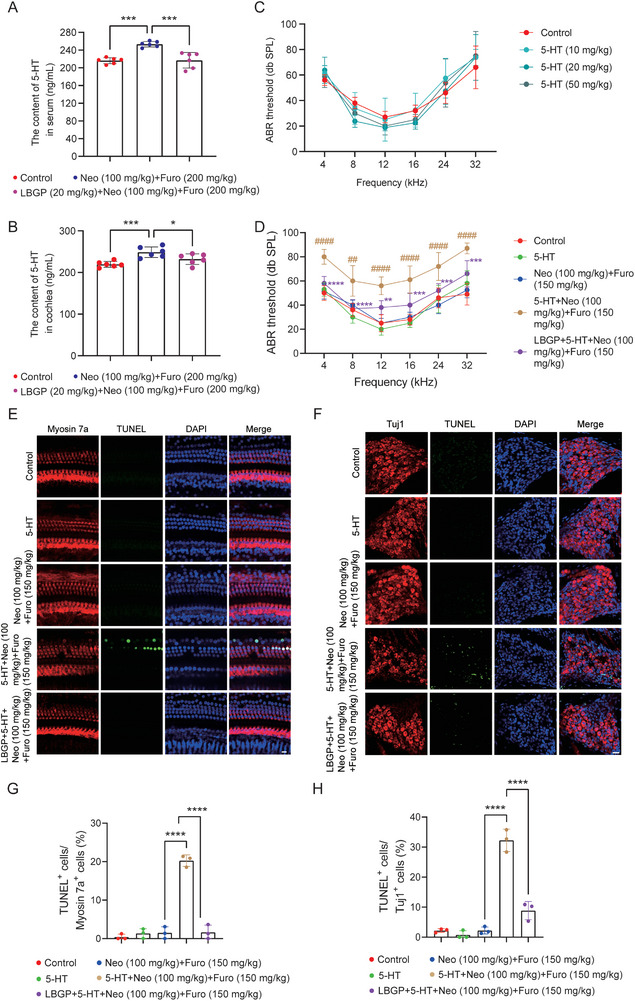
5‐HT exacerbates neomycin‐induced ototoxicity in C57BL/6 mice. A,B) The concentrations of 5‐HT in serum and the cochleae as determined by ELISA kits in the different groups (*n* = 6). Results are presented as mean ± SD, *P < 0.05, ***P < 0.001 by one‐way ANOVA with Bonferroni post hoc test. C) ABR analysis of hearing thresholds of mice after intravenous injection of 5‐HT at different doses (*n* = 6). D) ABR analysis of auditory function in the different groups (*n* = 6). Results are presented as mean ± SD, ^##^ *P* < 0.01, ^####^ *P* < 0.0001 versus Neo (100 mg kg^−1^)+Furo (150 mg kg^−1^) group; **P < 0.01, ***P < 0.001, **** *P* < 0.0001 versus 5‐HT+Neo (100 mg kg^−1^)+Furo (150 mg kg^−1^) group by two‐way ANOVA with Bonferroni post hoc test. E,F) TUNEL staining as an indicator of apoptosis in cochlear hair cells (stained with Myosin 7a) and SGNs (stained with Tuj1) (*n* = 3). Scale bar, 20 µm. G,H) Quantification of the proportions of TUNEL‐positive cochlear hair cells and SGN cells. Results are presented as mean ± SD, **** *P* < 0.0001 by one‐way ANOVA with Bonferroni post hoc test.

Next, we studied the effect of 5‐HT in neomycin‐induced ototoxicity. We first verified that neomycin could increase the level of 5‐HT both in serum and the cochlea, and there was a significant decrease in 5‐HT after LBGP treatment (**Figure** **5**A,B). To evaluate the effect of 5‐HT alone on hearing function, 5‐HT at different concentrations (10, 20, or 50 mg kg^−1^) was given by intravenous injection. ABR analysis showed that 5‐HT treatment alone had no influence on hearing function (Figure 5C). However, we observed that systemic administration of 5‐HT (50 mg kg^−1^) increased neomycin (100 mg kg^−1^) + furosemide (150 mg kg^−1^)‐induced hearing loss, which could be mitigated by LBGP treatment (Figure 5D). Immunostaining also showed that systemic administration of 5‐HT (50 mg kg^−1^) promoted neomycin (100 mg kg^−1^) + furosemide (150 mg kg^−1^)‐induced apoptosis of cochlear hair cells and SGNs, which also could be reversed by LBGP (Figure 5E–H). These results suggested that 5‐HT played a crucial role in neomycin‐induced hearing dysfunction.

### 2.6 Pharmacological Inhibition of Tph2 Alleviates Neomycin‐Induced Hearing Loss

The Tph2 enzyme plays a crucial role in the biosynthesis of 5‐HT. In order to better understand the impact of Tph2 on neomycin‐induced hearing loss, the Tph inhibitor PCPA was utilized in vivo to study the effects of Tph2 on hearing loss caused by neomycin (Figure S4A, Supporting Information). The experiment demonstrated a clear enhancement in auditory function following PCPA treatment, as indicated by Figure S4B (Supporting Information). Additionally, the number of surviving hair cells was notably increased, signifying a partial restoration in hearing capabilities as shown in Figure S4C–F (Supporting Information), indicating that PCPA administration had a protective effect on neomycin‐induced hearing loss. We then determined the effect of PCPA on cultured cochlear explants in vitro. We discovered that a concentration of PCPA at 0.5 µm effectively reduced the hair cell injury caused by neomycin in the basal and middle turns of the cultured explants (Figure S5A–D, Supporting Information). The loss of cochlear hair cells may be the result of apoptosis, so immunostaining of the apoptosis marker cleaved caspase 3 and a TdT‐mediated dUTP nick‐end labeling (TUNEL) assay were conducted to visualize the apoptotic cells in the cultured explants. As expected, PCPA treatment effectively ameliorated neomycin‐induced apoptosis in hair cells (Figure S5E–H, Supporting Information). These results showed that PCPA could partially alleviate neomycin‐induced ototoxicity by inhibiting apoptotic processes.

### 2.7 Tph2 Knock Down by Adeno‐associated Virus (AAV)‐Inner Ear (ie)‐Sh*Tph2* in Cochlear Hair Cells and SGNs Improves Hearing Function after Neomycin Exposure

**Figure 6:**
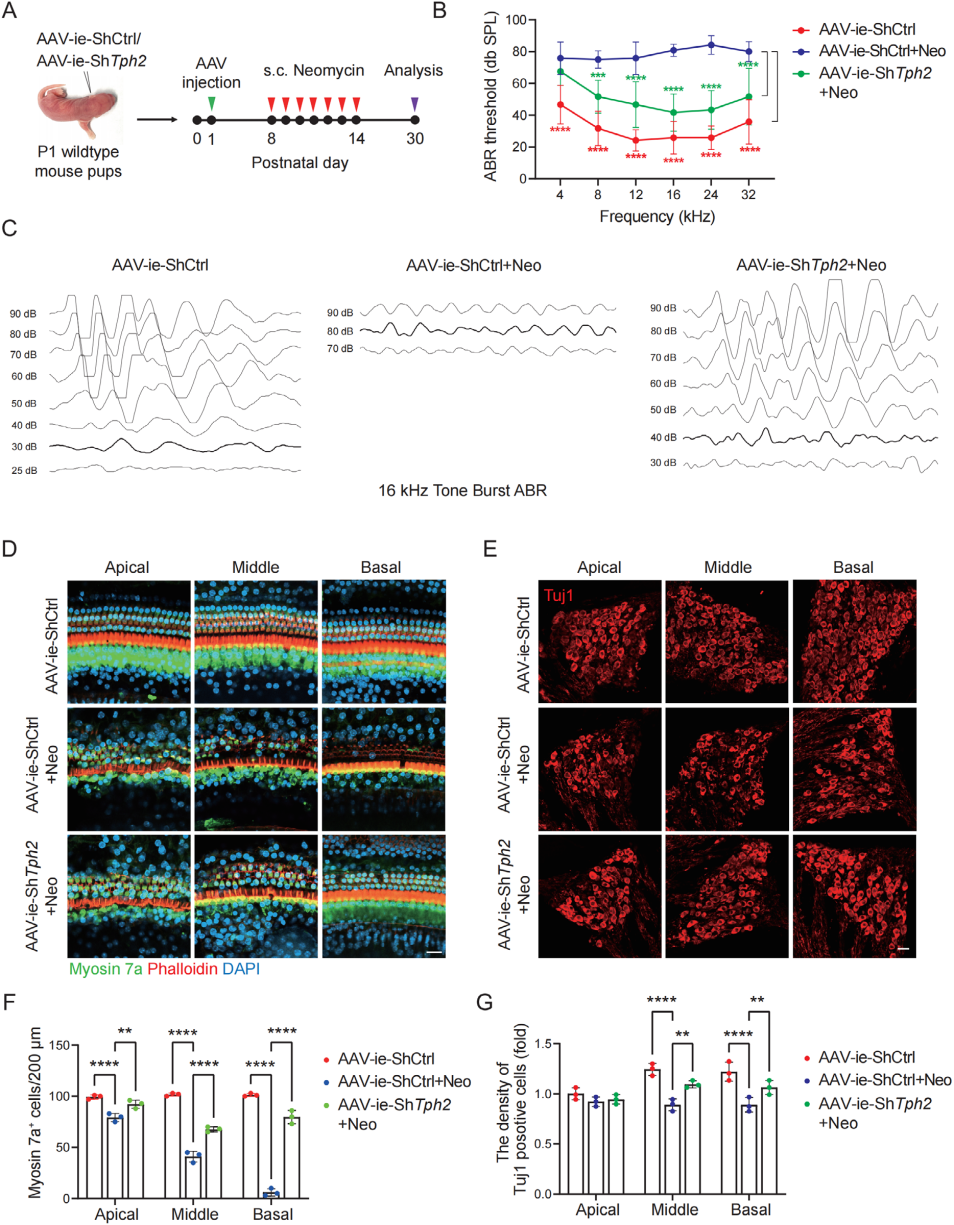
AAV‐ie‐Sh*Tph2* attenuates auditory impairment after neomycin treatment in FVB mice. A) Schematic illustration of the experimental design. B) ABR analysis for determine the hearing thresholds of different groups (*n* = 6). Results are presented as mean ± SD, ***P < 0.001, ****P < 0.0001 by two‐way ANOVA with Bonferroni post hoc test. C) Representative ABR waveforms in response to 16 kHz pure tone stimulation in the different groups. D) Immunostaining of cochlear hair cells with Myosin 7a and Phalloidin in the apical, middle, and basal turns in the different groups (*n* = 3). Scale bar, 20 µm. E) Immunostaining of cochlear SGNs with Tuj1 in the apical, middle, and basal turns in the different groups (*n* = 3). Scale bar, 20 µm. F,G) Quantification of Myosin 7a‐positive hair cells and Tuj1‐positive SGNs in the different groups. Results are presented as mean ± SD, **P < 0.01, ****P < 0.0001 by two‐way ANOVA with Bonferroni post hoc test.

We next decreased the endogenous expression of *Tph2* in P1 mouse cochleae by cochlear injection of AAV‐ie‐Sh*Tph2*. We constructed three Sh*Tph2* plasmids, and Sh*Tph2*‐2 exhibited the best knock down effect according to the western blot result (Figure S6A, Supporting Information). The Sh*Tph2‐2* plasmid was cloned into the AAV plasmid (Figure S6B, Supporting Information), and the AAV viruses were produced in HEK‐293T cells. Immunofluorescence results showed that AAV‐ie could effectively infect hair cells and SGNs in mouse cochleae (Figure S6C,D, Supporting Information) and that the expression of Tph2 in hair cells and SGNs was successfully knocked down by cochlear injection of AAV‐ie‐Sh*Tph2* (Figure S6E,F, Supporting Information). A chronic model of neomycin‐induced ototoxicity was constructed to evaluate the effect of AAV‐ie‐Sh*Tph2* on neomycin‐induced hearing impairment (**Figure** **6**A). The ABR assay showed that AAV‐ie‐Sh*Tph2* partially improved neomycin‐induced hearing loss at 8, 12, 16, 24, and 32 kHz (Figure 6B,C). And immunofluorescence analysis showed that AAV‐ie‐Sh*Tph2* partially promoted the survival of cochlear hair cells and SGNs after neomycin damage (Figure 6D–G). Taken together, these results showed that *Tph2* knocked down in the cochlea protected against neomycin‐induced hearing dysfunction.

## 3 Discussion

In the present study, we have identified a previously unrecognized role for LBGP in preventing neomycin‐induced hearing loss. We found that intragastric administration of LBGP effectively attenuated neomycin‐induced loss of cochlear hair cells, SGNs, neurofilaments, and ribbon synapses, all of which are crucial for the perception and transduction of sound signals. The neomycin‐induced redox imbalance in the cochlea was also improved by LBGP treatment. The metabolomics analysis showed that Tph‐mediated 5‐HT biosynthesis played an important role in the mechanism of action of LBGP in treating neomycin‐induced hearing impairment. In addition, pharmacological inhibition of Tph2 by PCPA or knockdown by AAV‐ie‐Sh*Tph2* significantly protected against neomycin‐induced auditory dysfunction.

*Lycium barbarum* polysaccharides are the biologically active components of the traditional Chinese herbal medicine *Lycium barbarum* which is commonly used to treat kidney and the liver injury, aging, diabetes, retinal degenerative diseases and neural disorders.^[^ ^17^, ^18^, ^19^, ^20^, ^21^, ^22^ ^]^ Meanwhile, according to the recordation in ancient medical books and Chinese pharmacopoeia in China, *Lycium barbarum* exhibits improvement on hearing function. The contents of *Lycium barbarum* polysaccharides are important for the efficacy of *Lycium barbarum*, but the biological functions of *Lycium barbarum* polysaccharides are complicated and multifaceted due to their complex composition. LBGP is further separated and purified from *Lycium barbarum* polysaccharides and shows stronger bioactivities and milder side effects.^[^ ^23^ ^]^ Numerous studies have reported that LBGP exerts significant neuroprotective effects and improves symptoms of depression, anxiety, and Parkinson's disease.^[^ ^9^, ^24^, ^25^ ^]^ Here we provide the first line of evidence that LBGP pretreatment can attenuate aminoglycoside‐induced ototoxicity, suggesting that LBGP might be developed as an effective food supplement and potential drug against hearing damage.

Aminoglycosides‐induced oxidative stress contributes to the destruction of cellular structures and caspase‐mediated apoptosis and is closely associated with disorders of the neurological system and the cardiovascular system.^[^ ^26^, ^27^, ^28^ ^]^ Multiple studies have shown that oxidative stress plays a key role in the pathogenesis of drug‐related hearing loss.^[^ ^29^, ^30^, ^31^ ^]^ Oxidative stress induced by an excess of ROS and decreased levels of antioxidant enzymes leads to the peroxidation of cell components and results in cell death in the cochlea.^[^ ^32^, ^33^ ^]^ Therefore, enhancing the activities of antioxidant enzymes, eliminating the accumulation of ROS, improving oxidative stress injury and maintaining redox balance are of significant importance for protecting against hearing loss.^[^ ^34^ ^]^ In the current study we observed that LBGP could significantly restore the neomycin‐induced decreased level of antioxidant genes‐*Sod1*, *Nqo1*, and *Gsr*. 4‐HNE is a byproduct of lipid peroxidation that has been linked to the development of pathological conditions in the body due to oxidative stress. It is believed to play a crucial role in the process of cell death induced by oxidative stress.^[^ ^35^ ^]^ 3‐NT is a versatile oxidative stress biomarker generated from the nitration of protein‐bound and free tyrosine residues,^[^ ^36^ ^]^ and elevated expression of 3‐NT has been reported in neurological disorders.^[^ ^37^ ^]^ Here we observed that neomycin treatment remarkably increased the expression of 4‐HNE and 3‐NT in the cochlea, while LBGP effectively inhibited the elevated expression of 4‐HNE and 3‐NT, indicating that LBGP might act as an antioxidant agent for treating neomycin‐induced ototoxicity.

Tryptophan is a nutritionally essential amino acid that plays a substantial role in maintaining normal physiologic functions in neuropsychiatric health, oxidative systems, inflammatory responses, and gastrointestinal health.^[^ ^38^ ^]^ In this work we found that LBGP administration significantly regulated tryptophan metabolism both in the serum and in the cochlea. Tryptophan is metabolized via three distinct pathways: the kynurenine pathway, the 5‐HT pathway, and the indole pathway.^[^ ^39^ ^]^ The kynurenine pathway plays a major role in inflammatory signaling. Metabolite ratios from this pathway enable insights into enzyme activities and immune activation.^[^ ^40^ ^]^ Indols are direct signaling molecules used by the microbiome in communication with the host.^[^ ^41^ ^]^ 5‐HT which acts as an important neurotransmitter is synthesized through the 5‐hydroxytryptophan pathway and functions as a mediator of multiple neurological diseases.^[^ ^42^, ^43^, ^44^ ^]^

Plenty of studies demonstrated that 5‐HT pathway played an important role in the auditory function.^[^ ^45^, ^46^, ^47^ ^]^ A clinical study found that the plasma level of 5‐HT was elevated in patients with sensorineural hearing loss (SNHL) and that 5‐HT might appear as a biomarker of SNHL.^[^ ^48^ ^]^ It also has been reported that 5‐HT 2B receptors are upregulated in age‐related hearing loss.^[^ ^49^ ^]^ These evidences suggest a critical role for 5‐HT in the pathogenesis of hearing impairment. As was reported that blood constitutes an important source of 5‐HT to the cochlea and elevated 5‐HT expression can promote oxidative stress in tissues,^[^ ^50^, ^51^ ^]^ we observed that 5‐HT levels were significantly elevated both in the serum and the cochlea after neomycin damage, which could be suppressed by LBGP treatment. 5‐HT is regulated by two isoforms of the rate‐limiting enzyme Tph. The peripheral synthesis of 5‐HT is initiated by Tph1, which is localized predominantly in gastrointestinal enteroendocrine cells, while cerebral 5‐HT is regulated by Tph2, which is found mainly in the brain.^[^ ^52^, ^53^ ^]^ We furthermore verified that the cochlear Tph expression was remarkably increased after neomycin damage, while there was a reduction after LBGP treatment. Supplement of 5‐HT alone by tail intravenous injection in mice did not impact hearing function, but aggravating neomycin‐induced hearing loss, further suggesting that 5‐HT played an important role in neomycin‐induced ototoxicity. Inhibition of Tph2 by PCPA or AAV‐ie‐Sh*Tph2* both mitigated neomycin‐induced hearing loss. Our data revealed that Tph‐mediated 5‐HT biosynthesis is of vital importance in the mechanism of action in neomycin‐induced auditory dysfunction and supposed that Tph2 might act as a potential pharmacological target for the treatment of aminoglycoside‐induced ototoxicity.

## 4 Conclusion

In summary, our study found that LBGP could effectively protect against neomycin‐induced hearing impairment and prevent cochlear oxidative stress. The underlying mechanism responsible for the ototoxicity induced by neomycin may be connected to the inhibition of Tph‐mediated 5‐HT biosynthesis. This discovery offers fresh perspectives on the potential pathways involved in neomycin‐induced hearing loss and suggests that LBGP could be a beneficial treatment for aminoglycoside‐induced SNHL.

## 5 Experimental Section

### Materials and Reagents

Neomycin (N6386) and furosemide (BP547) were purchased from Sigma (St. Louis, MO, USA). P‐chlorophenylalanine (PCPA) (HY‐B1368) was obtained from MedChemExpress (NJ, USA). Serotonin (T2209) was purchased from Topscience (Shanghai, China). Anti‐Myosin 7a (ab150386), anti‐Tph2 (ab184505), anti‐beta III Tubulin (ab52623), anti‐3‐Nitrotyrosine (ab110282), and anti‐Ctbp2 (ab128871) were obtained from Abcam (Cambridge, MA, USA). Alexa Fluor 488/594 Phalloidin (A12379/A12381) and anti‐Tph1 (PA1‐777) were obtained from Invitrogen (Waltham, MA, USA). DAPI solution (C0060) was purchased from Solarbio (Beijing, China). Anti‐NF‐200 (60331‐1‐Ig) was obtained from Proteintech (Wuhan, China). Anti‐Glur2 antibody (MAB397) was purchased from Merck Millipore (Darmstadt, Germany). Anti‐4‐Hydroxynonenal antibody (MA5‐27570) was got from Thermo Fisher Scientific (Waltham, MA, USA). Anti‐Cleaved Caspase 3 (CASP 3) antibody (9664S) was obtained from Cell Signaling Technology (Boston, MA, USA). Goat anti‐Rabbit/Mouse IgG (H+L) Cross‐Adsorbed Secondary Antibody, Alexa Fluor 488 (A‐11008/ A‐11001) and Goat anti‐Rabbit/Mouse IgG (H+L) Cross‐Adsorbed Secondary Antibody, Alexa Fluor 594 (A‐11012/A‐11005) were obtained from Invitrogen (Waltham, MA, USA). The TUNEL Assay Kit (A112‐01) was purchased from Vazyme (Nanjing, China), and the 5‐HT ELISA kit (ml001891) was obtained from Shanghai Enzyme‐linked Biotechnology (Shanghai, China).

### Preparation of LBGP

LBGP was supplied by the Ningxia Tianren Goji Biotechnology Co., Ltd. (Ningxia, China). The preparation of LBGP is described previously.^[^ ^9^, ^11^ ^]^ In brief, *Lycium barbarum* polysaccharides were isolated from *Lycium barbarum* by water extraction followed by ethanol precipitation. LBGP was then separated and purified by gel chromatography and ion exchange chromatography. The purity of LBGP was 93%, the structure and the HPLC profiles of LBGP was shown in Figure S1 (Supporting Information). The analytical conditions of HPLC are as follows: 1) Chromatographic column: gel column SK gelG3000 PWXL, 300*7.8 mm, 6 µm. 2) Mobile phase: phosphate buffer solution (0.05 mol L^−1^ Na_2_HPO_4_). 3) Flow rate: 0.5 mL min^−1^. 4) Column temperature: 30 °C. 5. Wavelength: 260 nm. 6. Sample size: 20 µL.

### Animal Experiments

The animal procedures in the study were conducted in accordance with ethical standards set by the Institutional Animal Care and Use Committee of Southeast University (20210606001) and carried out in compliance with the ARRIVE guidelines. Male C57BL/6 mice and FVB neonatal mice (postnatal day (P) 1) were obtained from Gempharmatech Co., Ltd. (Nanjing, China). 4‐week‐old C57BL/6 mice were given LBGP (5, 10, or 20 mg kg^−1^) by intragastric administration for 2 weeks, while the normal saline solution was given to the mice in the control and model groups by oral gavage. The acute hearing loss model was then established by intraperitoneal injection of neomycin (Sigma, N6386) at 100 mg kg^−1^ followed by the intraperitoneal injection of 200 mg kg^−1^ furosemide (Sigma, BP547) 1 h later. Hearing function was measured after mice were anesthetized with pentobarbital (1%) 7 d later. To assess the protective effect of AAV‐Sh*Tph2* on chronic models^[^ ^3^, ^54^ ^]^ of ototoxicity, the left ears of FVB mouse pups (P1) were injected with 2 µL AAV‐ie‐Sh*Tph2* once through the semicircular canal. Seven days after injection, neomycin (200 mg kg^−1^) was injected subcutaneously for seven consecutive days and hearing function was determined at P30. Tissue collection: After rapidly dissecting the temporal bones of mice in cold PBS, the cochleae were fixed overnight in 4% paraformaldehyde at 4 °C and decalcified the next day in 10% EDTA at room temperature for 1 d. The decalcified cochlea was dissected using a microscope to isolate the basement membrane.

### ABR Audiometry

ABR was employed to determine the auditory function of mice as previously described.^[^ ^55^ ^]^ Briefly, after being anesthetized with pentobarbital sodium (1%), mice were placed on a preheated pad (37 °C). To record the electric response of mice, three subcutaneous needle electrodes were placed—with the main active electrode inserted at the vertex and the reference electrodes inserted below the ears. Different frequencies (4, 8, 12, 16, 24, and 32 kHz) were used for the ABR test using a Tucker‐Davis Technologies System.

### Cochlear Explant Culture

The cochleae of P2 FVB mice were dissected and cultured as previously reported.^[^ ^56^ ^]^ In brief, the cochleae were rapidly isolated in cold HBSS (Multicell, 311512011) and attached to Cell‐Tak (Corning, 354240)‐coated slides. The explants were cultured in DMEM‐F12 medium containing 1% N2 supplement (Gibco, A1370701), 2% B27 supplement (Gibco, 17504044) and 0.1% ampicillin (Beyotime, ST008). The next day PCPA was added to the cochlear explants for incubation for 12 h at 37 °C, then neomycin (0.5 mm) together with PCPA was administrated for 24 h.

### Immunofluorescence

The cochleae that were isolated underwent fixation with 4% PFA and decalcification using 0.5 m EDTA for 48 h. Following permeabilization with a solution of 1% Triton X‐100/PBS for 30 min and subsequent blocking with 10% donkey serum for 1 h at room temperature, the specimens were subjected to overnight incubation with primary antibodies at 4 °C. Subsequently, the specimens underwent triple washing with PBS and incubation with relevant secondary antibodies at room temperature for 1 h on the following day. After another round of triple PBS wash, the samples underwent imaging through a confocal laser scanning microscope (Zeiss, Germany). Quantification of SGNs: The number of Tuj1 positive cells in the same acreage (100 µm×100 µm) of the samples from different groups was counted and the values of all groups were normalized to the control group.

### qPCR

RNA was extracted from the cochleae of mice using the FastPure Cell/Tissue Total RNA Isolation Kit (Vazyme, RC112‐01). The extracted RNA was then transcribed into cDNA using the HiScript III first Strand cDNA Synthesis Kit (Vazyme, R312‐01). Subsequently, qPCR analysis was carried out utilizing the AceQ universal SYBR qPCR Master Mix (Vazyme, Q511‐02) on the CFX96 real‐time PCR system (Bio‐Rad, Hercules, CA, United States). GAPDH served as the control within the same samples, and data analysis was performed using the comparative cycle threshold (ΔΔCt) method.

### Enzyme‐Linked Immunosorbent Assay (ELISA)

The ELISA kit was used to determine the levels of 5‐HT in both serum and cochlea, following the manufacturer's instructions. The samples were added to the ELISA plates after diluting five‐fold. After incubation at 37 °C for 30 min, the samples were washed with wash buffer for 30 s and patted dry followed by washing five times. The HRP‐conjugate reagent was added to each well followed by incubation and washing. The chromogenic solution was added to each well of the ELISA plates for 15 min at 37 °C in dark, and the level of 5‐HT was measured at 450 nm wavelength using a Thermo Scientific microplate reader.

### TUNEL Staining

The apoptosis of cochlear hair cells and SGNs was detected using a TUNEL Assay Kit following the manufacturer's recommendations. In short, the specimens underwent three washes in a PBS solution, followed by equilibration with Equilibration buffer for 15 min at room temperature. Next, the specimens were labeled with labeling buffer for 1 h at 37 °C in darkness and subsequently rinsed twice with PBS. Finally, visualization was carried out using a confocal laser scanning microscope (Zeiss, Germany).

### Nontargeted Metabolomics

Following relocation to a 1.5 mL Eppendorf tube, the specimens were agitated using a vortexer for a duration of 10 s. L‐2‐chlorophenylalanine was used as the internal standard in a concentration of 0.3 mg mL^−1^ and dissolved in methanol. This mixture was then combined with a pre‐cooled mixture of methanol and acetonitrile, and vortexed for 1 min. This method of using L‐2‐chlorophenylalanine as the internal standard in methanol proved to be effective in this experiment. By combining it with the mixture of methanol and acetonitrile and vortexing, a homogenous solution was achieved, ensuring accurate and precise results in the analysis. This step is crucial in maintaining consistency and reliability in the research process. Following the extraction of the samples via ultrasonication in an ice‐water bath for 10 min and subsequent storage at ‐20 °C for 30 min, they underwent centrifugation at 13000 ×*g* at 4 °C for 10 min, resulting in the drying of the supernatant. To ensure quality control, a pooled sample was prepared by combining portions of all samples. Subsequently, the addition of methoxylamine hydrochloride in pyridine, followed by vortexing for 2 min and an incubation at 37 °C for 90 min, took place. BSTFA and n‐hexane were brought to the mixture, which was then vortexed for 2 min and derivatized at 70 °C for 1 h. The processed specimens were subjected to analysis using an Agilent 7890B gas chromatography system linked to an Agilent 5977A MSD system (Agilent Technologies Inc., CA, USA).

### Data Processing

The data were imported into software MS‐DIAL, which performs peak detection, peak identification, MS2Dec deconvolution, characterization, peak alignment, wave filtering, and missing value interpolation. Metabolite characterization is based on LUG database. A data matrix was derived. The 3D matrix includes: sample information, the name of the peak of each substance, retention time, retention index, mass‐to‐charge ratio, and signal intensity. In each sample, all peak signal intensities were segmented and normalized according to the internal standards with RSD greater than 0.3 after screening. After the data was normalized, redundancy removal and peak merging were conducted to obtain the data matrix. The matrix was imported in R to carry out principle component analysis (PCA) to observe the overall distribution among the samples and the stability of the whole analysis process. Orthogonal partial least‐squares‐discriminant analysis (OPLS‐DA) and partial least‐squares‐discriminant analysis (PLS‐DA) were utilized to distinguish the metabolites that differ between groups.

### AAV‐ie Virus Preparation

The AAV plasmid containing the CAG promoter and the WPRE cassette had the Tph2 transgene plasmid integrated, with AAV2 inverted terminal repeats flanking it. The AAV viruses were generated by transfecting HEK‐293T cells (ATCC) with a rep‐cap fused plasmid, a helper plasmid, and the transgene plasmid. This process allowed for the production of the desired AAV viruses for further studies and experiments to be conducted. AAVs were purified as previously reported,^[^ ^57^ ^]^ and the AAVs titers were determined by SYBR analysis using primers for the WPRE region: Forward, 5′‐GTCAGGCAACGTGGC GTGGTGTG‐3′; Reverse, 5′‐GGCGATGAGTTCCGCCGTGGC‐3′.

### Statistical Analysis

All data in this study were presented as mean ± standard deviation (SD) from at least three independent experiments and *n* represents the number of samples from each subgroup. All data were analyzed with GraphPad Prism software. Statistical significance was calculated with Student's t‐test for comparisons between two groups or one‐way analysis of variance (ANOVA) for multiple comparisons followed by the Bonferroni post hoc test. The ABR results were evaluated with two‐way ANOVA. Differences were considered significant at *P* values < 0.05.

## Conflict of Interest

The authors declare no conflict of interest.

## Author Contributions

Y.W., L.Z., and S.C. contributed equally to this work. Y.W., L.Z., K.F.S., and R.C. designed the study. Y.W., L.Z., and S.C. carried out the experiments. J.Z., Q.L., and Z.Y. analyzed the data. Q.F., Y.Z., and C.L. performed part of the experiments. Y.W. and X.F. wrote the manuscript. K.‐F.S. and Y.S. reviewed and prepared the final version of the manuscript. All authors discussed the idea and approved the final version.

## Supporting information



Supporting Information

## Data Availability

The data that support the findings of this study are available from the corresponding author upon reasonable request.
