# Correction to “*Lycium barbarum* Glycopeptide Alleviates Neomycin‐Induced Ototoxicity by Inhibiting Tryptophan Hydroxylase‐Mediated Serotonin Biosynthesis”

**DOI:** 10.1002/advs.74866

**Published:** 2026-03-17

Wu Y, Zhang L, Cao S, Zhang J, Li C, Shan Y, Liu Q, Yu Z, Fang Q, Zhang Y, Fu X, So KF, Chai R. Lycium barbarum Glycopeptide Alleviates Neomycin‐Induced Ototoxicity by Inhibiting Tryptophan Hydroxylase‐Mediated Serotonin Biosynthesis. *Adv Sci (Weinh)*. 2025;12(29):e2405850, https://doi.org/10.1002/advs.202405850

In the version of this article initially published, we found that the images of the LBGP (10 mg/kg)+Neo+Furo group in Figure 3E were improperly used. The corrected figure is shown below.

Corrected Figure 3E:


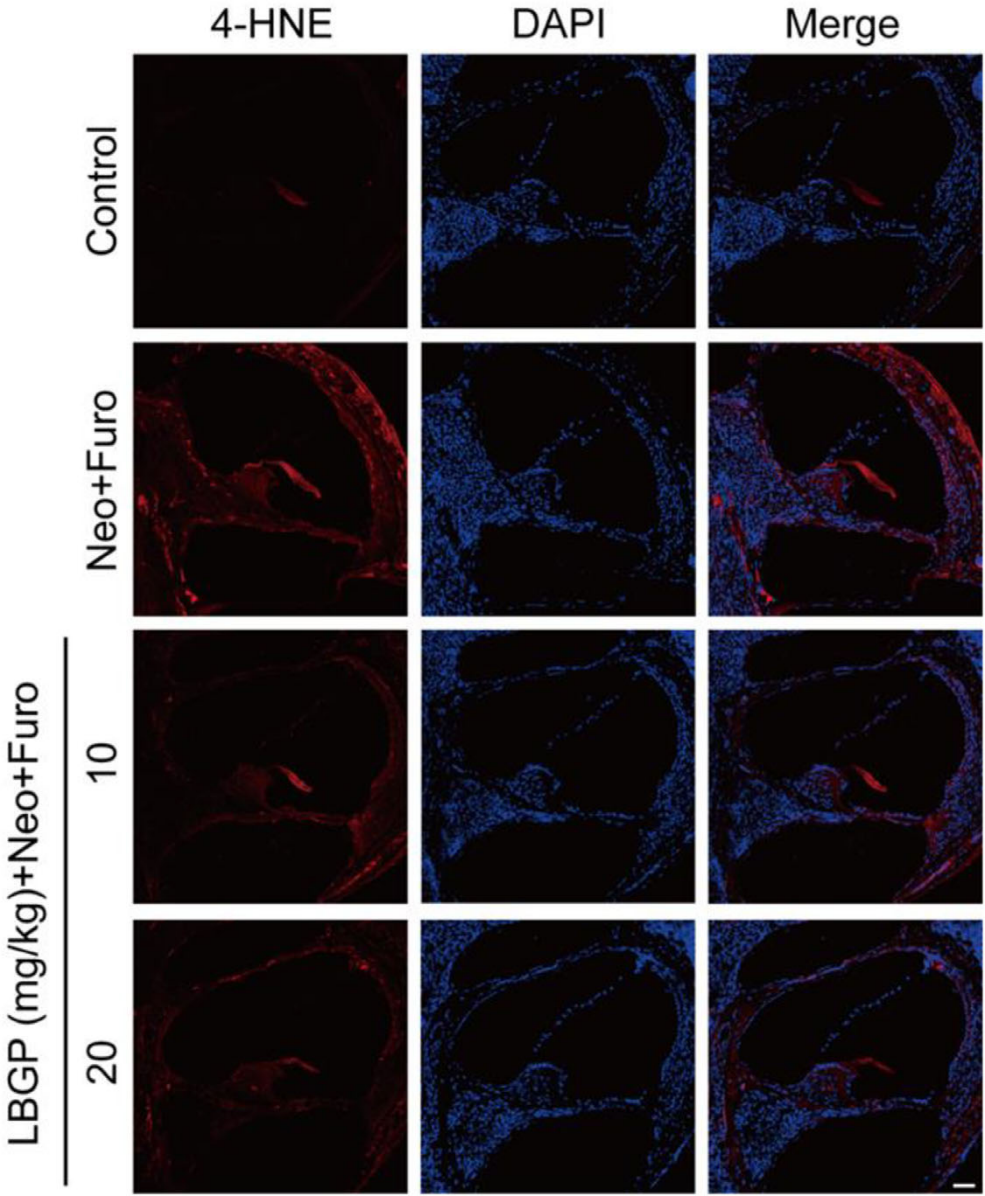


This correction does not affect the overall findings and conclusions of this paper. We apologize for this error.

