# Supplementary material for: Lycium barbarum Glycopeptide Alleviates Neomycin‐Induced Ototoxicity by Inhibiting Tryptophan Hydroxylase‐Mediated Serotonin Biosynthesis
Source: Adv Sci (Weinh). 2025 Mar 26;12(29):2405850. doi: 10.1002/advs.202405850 (PMC12362758; doi:10.1002/advs.202405850)
Supplement: Supplementary file 1 — Supporting Information [file ADVS-12-2405850-s001.docx]

Supporting Information

***Lycium barbarum* glycopeptide alleviates neomycin-induced ototoxicity by inhibiting tryptophan hydroxylase-mediated serotonin biosynthesis**

*Yunhao Wu, Li Zhang, Shengda Cao, Jingwen Zhang, Cheng Li, Yunlong Shan, Qiuping Liu, Zhexiong Yu, Qiaojun Fang, Yuhua Zhang, Xiaolong Fu^*^, Kwok-Fai So^*^, Renjie Chai^*^*

**
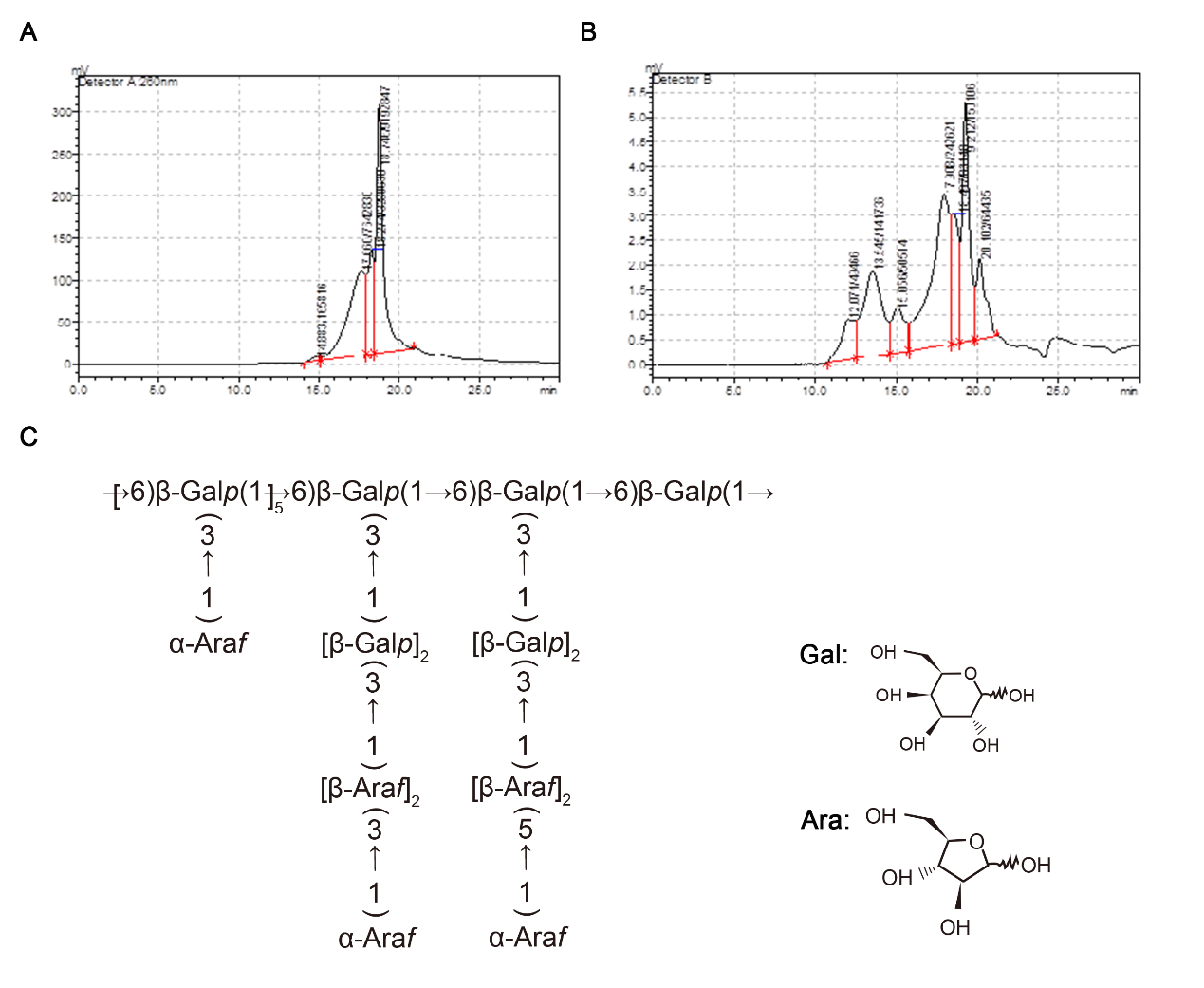
**

**Figure S1** HPLC profiles and structure of LBGP. (A) Specific chromatogram at UV 260nm. (B) Infrared-specific chromatogram. (C) The structure of the repeating unit of the glycan of LBGP.


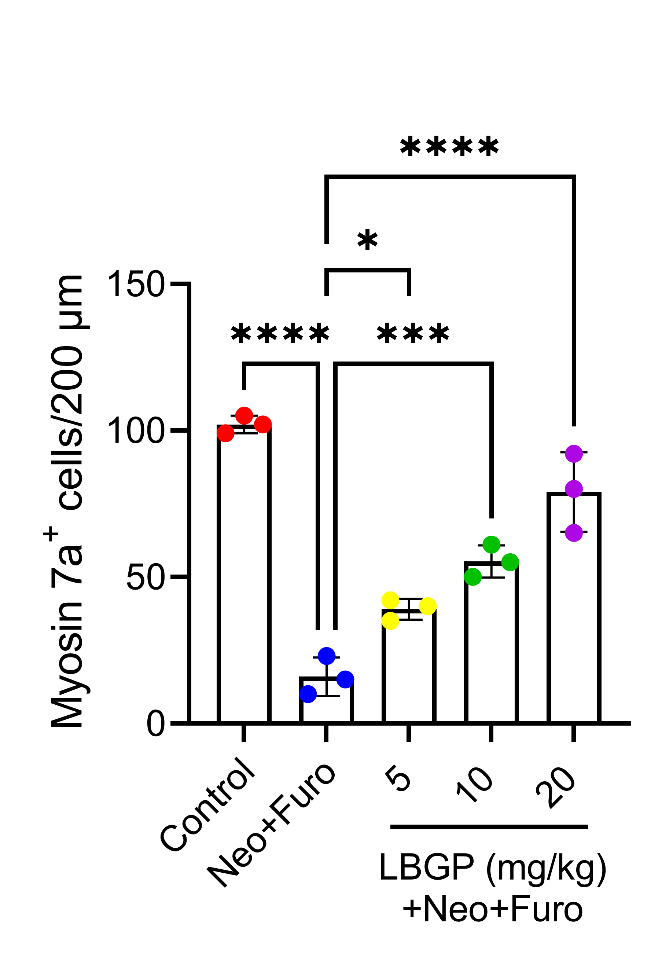


**Figure S2** Quantification of cochlear hair cell in Figure 1C. Results are presented as mean ± SD, * *P* < 0.05, *** *P* < 0.001, **** *P* < 0.0001 by one-way ANOVA with Bonferroni post hoc test.


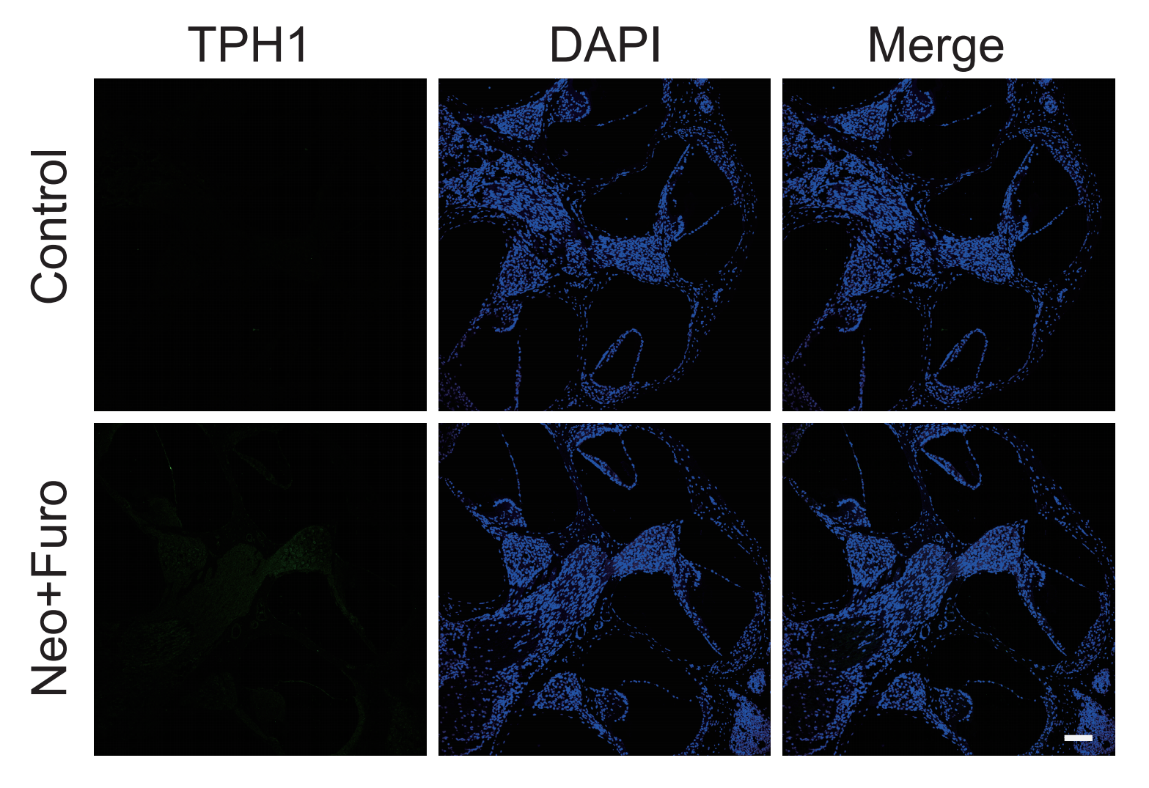


**Figure S3** Expression of TPH1 in C57BL/6 mouse cochlea in the control group and the neomycin treatment group by immunostaining (n = 3). Scale bar, 100 μm.


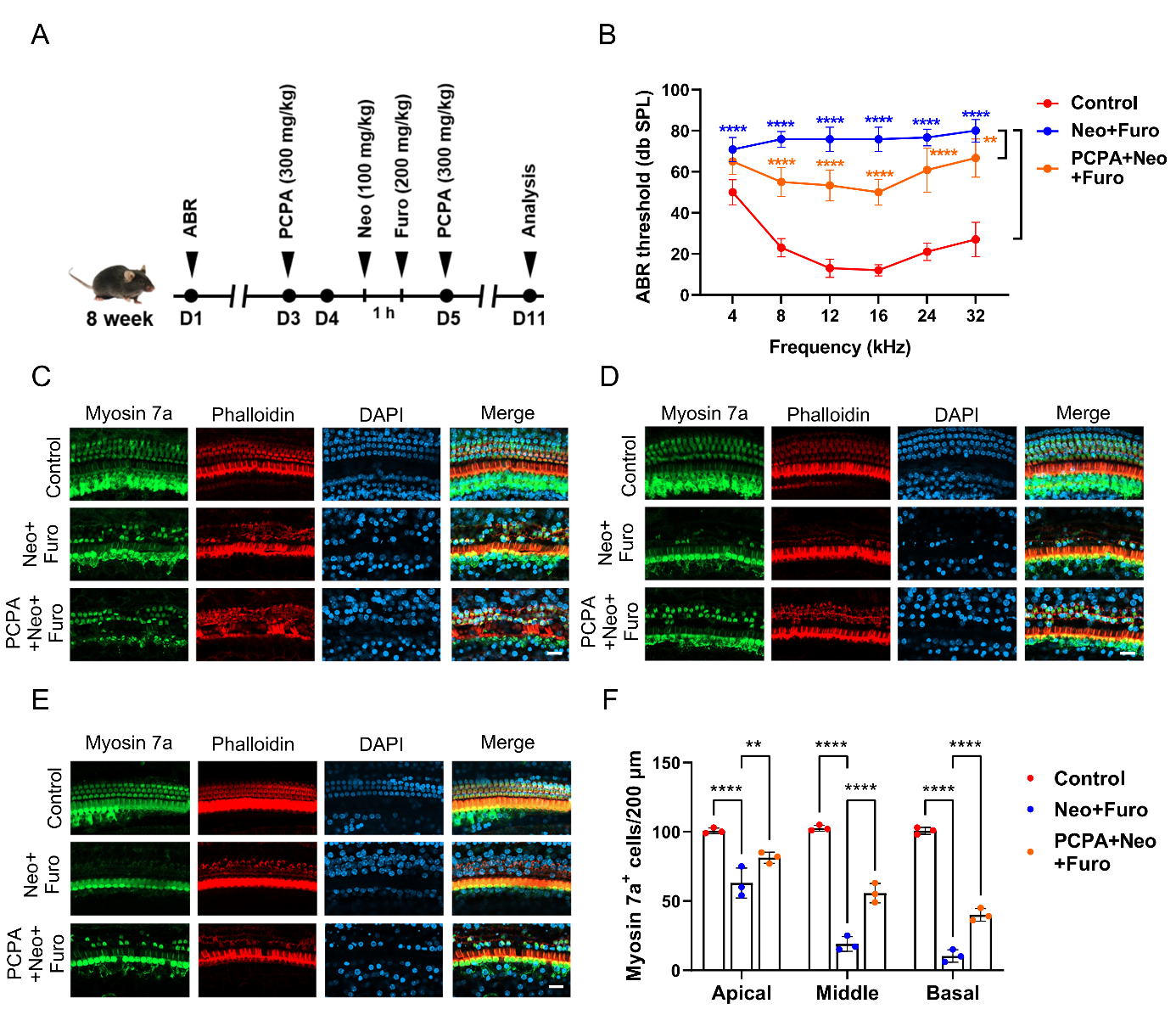


**Figure S4** PCPA restores neomycin-induced hearing dysfunction in C57BL/6 mice. (A) Schematic illustration of the experimental design. (B) ABR analysis for detecting hearing thresholds in the different groups (n = 6). Results are presented as mean ± SD, ***P* < 0.01, *****P* < 0.0001 by two-way ANOVA with Bonferroni post hoc test. (C-E) Immunostaining of cochlear hair cells with Myosin 7a and Phalloidin in the apical (C), middle (D), and basal (E) turns in the different groups (n = 3). Scale bar, 20 μm. (F) Quantification of Myosin 7a-positive hair cells in the apical, middle, and basal turns in the different groups. Results are presented as mean ± SD, ***P* < 0.01, *****P* < 0.0001 by two-way ANOVA with Bonferroni post hoc test.


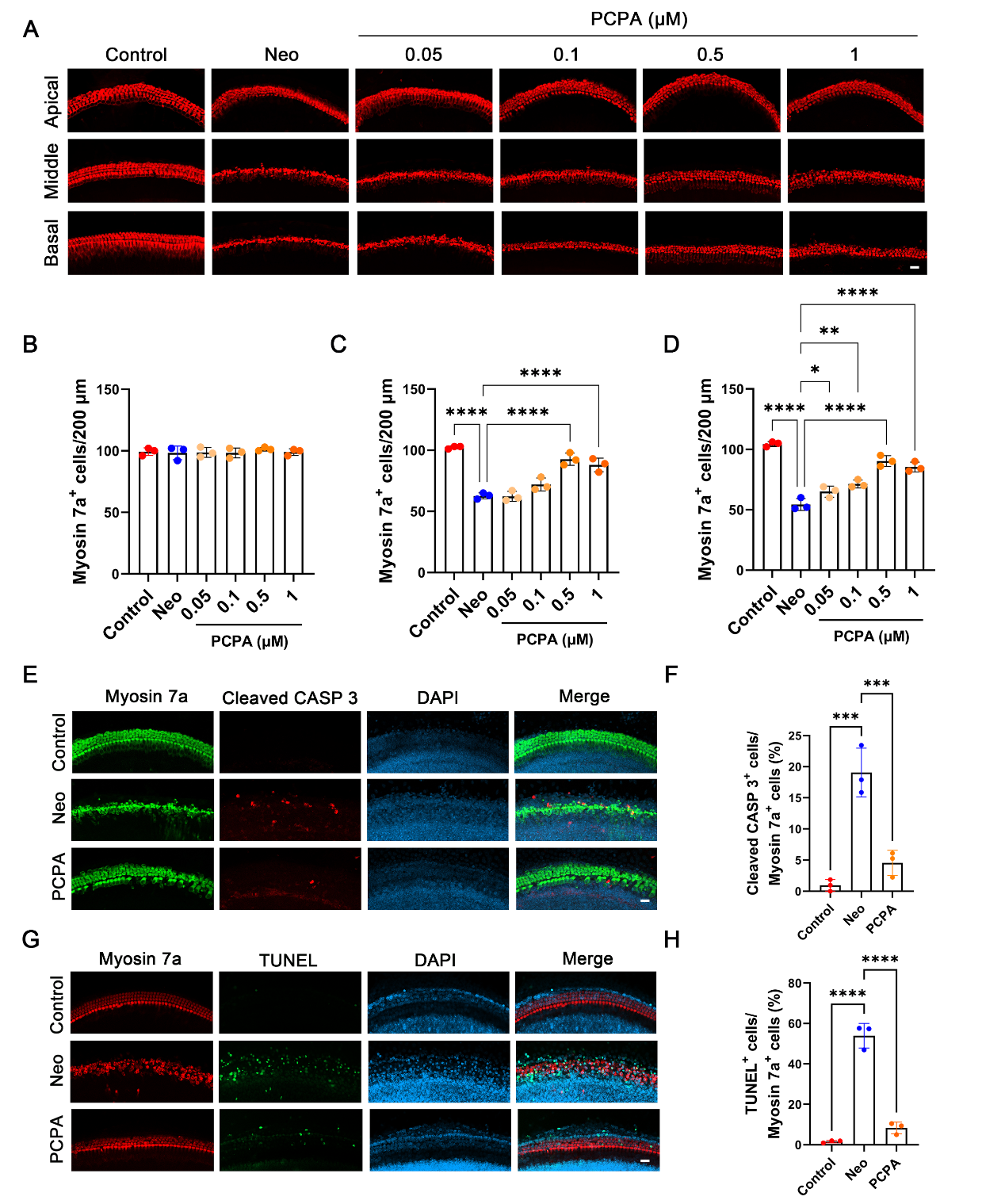


**Figure S5** PCPA alleviates neomycin-induced apoptosis of cochlear hair cells in cochlear explants. (A) Immunostaining of hair cells with Myosin 7a in cultured explants (n = 3). Scale bar, 20 μm. (B-D) Quantification of Myosin 7a-positive hair cells in the apical (B), middle (C), and basal (D) turns in the different groups. (E) Immunostaining of cleaved caspase 3 and Myosin 7a in cochlear hair cells in the middle turns in the different groups (n = 3). Scale bar, 20 μm. (F) Quantification of cleaved caspase 3-positive hair cells in (E). (G) Immunostaining of TUNEL and Myosin 7a in cochlear hair cells in the middle turns in the different groups (n = 3). Scale bar, 20 μm. (H) Quantification of cleaved caspase 3-positive hair cells in (G). Results are presented as mean ± SD, * *P* < 0.05, ** *P* < 0.01, *** *P* < 0.001, **** *P* < 0.0001 by one-way ANOVA with Bonferroni post hoc test.


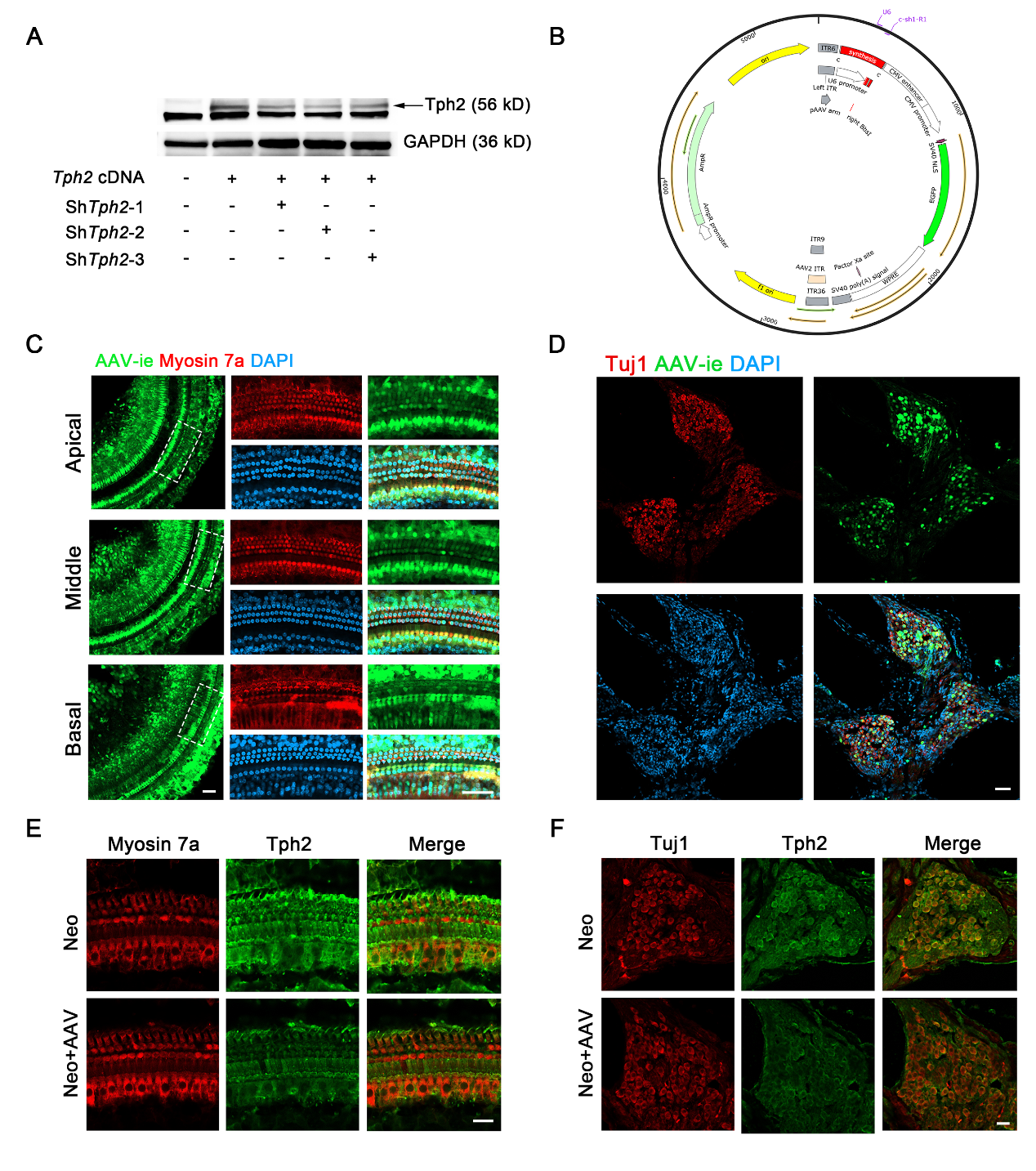


**Figure S6** Construction and verification of AAV-ie-Sh*Tph2* in FVB mice. (A) Expression of Tph2 in *Tph2* cDNA and sh*Tph2* plasmids co-transfected HEK-293T cells. (B) The plasmid profile of AAV-ie. (C) Representative images of the apical, middle, and basal turns of cochleae injected at P1 with AAV-ie. Hair cells were stained with Myosin 7a (n = 3). Scale bar, 50 μm. (D) Representative images of SGNs injected at P1 with AAV-ie. SGNs were stained with Tuj1 (n = 3). Scale bar, 50 μm. (E) Immunostaining of Myosin 7a and Tph2 in cochlear hair cells in the different groups (n = 3). Scale bar, 20 μm. (F) Immunostaining of Tuj1 and Tph2 in SGNs in the different groups (n = 3). Scale bar, 20 μm.
